# Slc39a5-mediated zinc homeostasis plays an essential role in venous angiogenesis in zebrafish

**DOI:** 10.1098/rsob.200281

**Published:** 2020-10-21

**Authors:** Zhidan Xia, Xinying Bi, Jia Lian, Wei Dai, Xuyan He, Lu Zhao, Junxia Min, Fudi Wang

**Affiliations:** The First Affiliated Hospital, School of Public Health, Institute of Translational Medicine, Zhejiang University School of Medicine, Hangzhou, People's Republic of China

**Keywords:** Slc39a5, zinc homeostasis, angiogenesis, zebrafish

## Abstract

Angiogenesis is a precise process mediated by a variety of signals and the environmental niche. Although the essential trace element zinc and its homeostasis are essential for maintaining proper cellular functions, whether zinc plays a role in angiogenesis is currently unknown. Using zebrafish embryos as a model system, we found that zinc treatment significantly increased the expression of the *slc39a5* gene, which encodes the zinc transporter Slc39a5. Moreover, knocking down *slc39a5* expression using either a morpholino or CRISPR/Cas9-mediated gene editing led to cardiac ischaemia and an accumulation of red blood cells in the caudal vein plexus (CVP), as well as delayed venous sprouting and fewer vascular loops in the CVP region during early development. Further analysis revealed significantly reduced proliferation and delayed cell migration in the caudal vein of *slc39a5* morphants. At the mechanistic level, we found increased levels of systemic zinc in *slc39a5*-deficient embryos, and chelating zinc restored CVP development. In addition, we found that zinc overload in wild-type embryos leads to impaired CVP formation. Taken together, these results indicate that Slc39a5 plays a critical role in endothelial sprouting and migration in venous angiogenesis by regulating zinc homeostasis.

## 1. Introduction

Angiogenesis is an essential biological process in which new blood vessels emanate from existing vascular structures, with vascular budding, migration, proliferation and pruning, thereby establishing the vascular network [1,2]. In zebrafish, the dorsal aorta and axial vein form a primitive circulatory loop, and angiogenesis from these vessels plays a key role in generating complex vascular networks. Specifically, the posterior axial vein extends ventrally at 26–32 hours post-fertilization (hpf), to eventually form a ‘honeycomb-like’ network at 38 hpf; this network, called the caudal vein plexus (CVP), is composed of a dorsal vein and a ventral vein, with interconnecting vessels [3].

Angiogenesis is necessary for the efficient delivery of oxygen and plays a critical role in a wide range of physiological and pathophysiological processes, including tissue development, nutrient transport, wound healing and tumour neovascularization [3–5]. However, the underlying cues and mechanisms that initiate and maintain angiogenesis are poorly understood; moreover, the role of zinc and zinc homeostasis in angiogenesis has not been investigated.

As an essential trace element, zinc is important for maintaining cellular physiology and for the development of various organs and tissues [6–8]. At the cellular level, zinc homeostasis is controlled by two families of transporter proteins, including 14 members of the SLC39A (Zrt- and Irt-like protein, ZIP) family and 10 members of the SLC30A (zinc efflux transporter, ZnT) family [9,10]. Interestingly, the zinc transporter SLC39A12 was reported to serve as a major regulator of pulmonary vascular remodelling under low-oxygen conditions, as genetically deleting *Slc39a12* expression protected against the development of pulmonary hypertension in rats housed in a hypoxic atmosphere [11]. However, whether other members of the SLC39A family play role in vascular development is an open question.

The zinc transporter SLC39A5, mediates the transport of metal ions across cell membranes, including the plasma membrane and subcellular organelles [12]. In intestinal enterocytes, SLC39A5 is expressed in the basolateral membrane and may play an important role in communicating the body's zinc status to enterocytes in order to control systemic zinc homeostasis [13,14]. In addition, SLC39A5 has been reported to play important roles under both physiological and pathological conditions. For example, β-cell-specific *Slc39a5* knockout mice have impaired insulin secretion and reduced glucose tolerance [15]. In oesophageal squamous cell carcinoma, *SLC39A5* is highly upregulated and may play a role in cellular proliferation and migration, promoting disease progression [16]. In addition, *SLC39A5* is expressed in high levels in the sclera and retina, and mutations in *SLC39A5* have been linked to autosomal dominant non-syndromic high myopia in humans [17]. Nevertheless, whether SLC39A5 and/or SLC39A5-mediated zinc homeostasis plays a role in vascular development or angiogenesis is currently unknown.

Here, we report that SLC39A5 plays an important role in angiogenesis during early development in zebrafish. Specifically, we found that maintaining systemic zinc concentrations is essential for the proliferation and migration of endothelial cells during venous angiogenesis. These results reveal a new function for SLC39A5 in early vertebrate development with respect to zinc homeostasis during angiogenesis, providing important new insights into the signals and mechanisms that underlie this process and suggesting a potential new therapeutic target for treating angiogenesis-related diseases.

## 2. Results

### 2.1. Knocking down *slc39a5* leads to blood accumulation in the zebrafish caudal vein plexus

**Figure 1.**
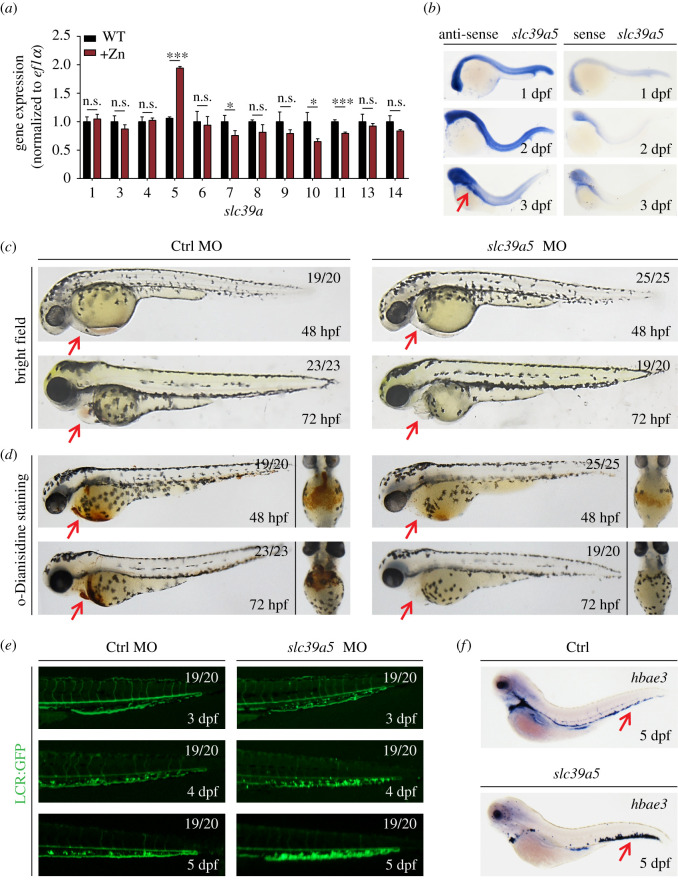
*slc39a5* morphant zebrafish embryos develop cardiac ischaemia and blood cell accumulation in the caudal vein plexus. (*a*) Quantitative PCR analysis of the 12 indicated *slc39a* genes measured in wild-type (WT) embryos and embryos treated with 1 mM zinc (+Zn). Note zebrafish lacking *slc39a2 and slc39a12*. (*b*) *In situ* hybridization of WT zebrafish embryos using the antisense and sense (control) *slc39a5* probes. Note the concentrated expression in the head and gut region at 3 dpf (red arrow). (*c*–*d*) Representative images (*c*) and o-Dianisidine-stained (*d*) control and *scl39a5* morphant embryos at the indicated developmental stages. The red arrows indicate the heart region. (*e*) Representative images of GFP fluorescence measured in the caudal region of control and *slc39a5* morphant Tg(globinLCR:eGFP) embryos. Note the accumulation of RBCs in the CVP of *slc39a5* morphants. (*f*) Whole-mount *in situ* hybridization of the RBC marker *hbea3* in control and *slc39a5* morphants. Note the increased signal in the CVP of the *slc39a5* morphant (arrow).

To determine whether members of the SLC39A family play a role in regulating systemic zinc homeostasis in zebrafish, we exposed wild-type (WT) embryos to 1 mM zinc and measured the expression of *slc39a1* through *slc39a14*. We found that zinc treatment significantly and selectively increased the expression of *slc39a5* (figure 1*a*). Using whole-mount *in situ* hybridization, we also found that *slc39a5* is expressed uniformly through WT zebrafish embryos at 1–2 days post-fertilization (dpf), and its expression becomes concentrated at the head and gut region by 3 dpf (figure 1*b*).

To analyse the functional role of Slc39a5 in zebrafish, we knocked down *slc39a5* expression using an ATG-based morpholino to prevent translation. We found that *slc39a5* morphants develop normally until 48 hpf, when they develop cardiac ischaemia (figure 1*c*). By 72 hpf, *slc39a5* morphants have severely reduced blood flow in the heart compared with control embryos (figure 1*c*). Using o-Dianisidine to stain haem confirmed the presence of cardiac ischaemia in *slc39a5* morphants at 48–72 hpf (figure 1*d*).

To test whether the cardiac ischaemia in *slc39a5* morphants was due to impaired cardiovascular function, we measured heart rate and cardiac morphology in control and morphant embryos. We found no significant difference between control and morphant embryos with respect to either heart rate (electronic supplementary material, figure S1*a*) or cardiac morphology (electronic supplementary material, figure S1*b*). To determine whether the *slc39a5* morphants develop anaemia, we measured red blood cells (RBCs) in the transgenic zebrafish line Tg(globinLCR:eGFP), in which RBC are fluorescent [18]. We found no significant difference in the number of RBCs between control and morphant embryos measured at 26–72 hpf (electronic supplementary material, figure S1*c–e*). Interestingly, however, we found that RBCs accumulated in the CVP of morphants but not in control embryos (figure 1*e*). This result was confirmed by performing *in situ* hybridization of *hbea3*, a marker for RBCs (figure 1*f*). Taken together, these results show that knocking down *slc39a5* in zebrafish embryos causes RBCs to accumulate in the CVP and reduces cardiac blood flow, resulting in cardiac ischaemia.

### 2.2. Slc39a5 morphants have delayed angiogenesis in the caudal vein plexus

**Figure 2.**
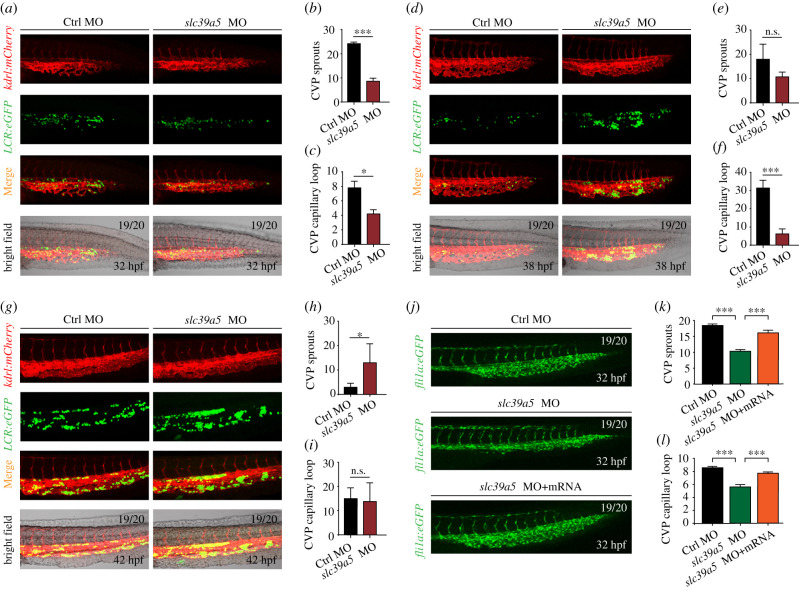
*slc39a5* morphants have delayed CVP development. (*a*–*g*) Representative images of the caudal region in control and *slc39a5* morphant embryos captured using dual-fluorescence microscopy at 32 hpf (*a*), 38 hpf (*d*) and 42 hpf (*g*). The numbers of endothelial sprouts and capillary loops in the CVP were measured at 32 hpf (*b*–*c*), 38 hpf (*e*–*f*) and 42 hpf (*h*–*i*). (*j*–*l*) Representative images of the CVP in control *Tg*(*fli1a*:eGFP) embryos and *Tg*(*fli1a*:eGFP) embryos injected with the *slc39a5* morpholino alone or co-injected with the *slc39a5* morpholino and a morpholino-resistant *slc39a5* mRNA. The numbers of endothelial sprouts (*k*) and capillary loops (*l*) in the CVP were measured at 32 hpf. *p < 0.05, ***p < 0.001 and n.s., not significant.

Next, we examined the vascular structure and blood distribution in the CVP using a dual-fluorescent zebrafish expressing the endothelial cell marker *kdrl*:mCherry [19] and globinLCR:eGFP. Compared with control embryos, *slc39a5* morphants had significantly fewer filopodia sprouting from exiting endothelial cells at 32 hpf (figure 2*a*,*b*), the age at which the caudal vasculature begins budding in the CVP [3]. Similarly, the *slc39a5* morphants had significantly fewer capillary loops compared with control embryos at 32 hpf (figure 2*a*,*c*). At 38 hpf, when the honeycomb-like network finally forms in the CVP of control embryos [3], the vascular plexus were considerably less developed in *slc39a5* morphants, with significantly fewer capillary loops (figure 2*d–f*). In addition, we found an increased number of RBCs in the caudal veins of *slc39a5* morphants compared with control embryos (figure 2*d*, green fluorescence), suggesting that the accumulation of RBCs in the CVP of morphant embryos is associated with the reduced development of caudal veins. At 42 hpf, control embryos no longer had CVP sprouts, whereas a significantly higher number of sprouts were present in the caudal veins of *slc39a5* morphants (figure 2*g–i*), indicating delayed venous angiogenesis in the CVP region.

To determine whether impaired venous angiogenesis in *slc39a5* morphants is tissue-specific, we analysed the development of the sub-intestinal vein plexus (SIVP), which sprouts from the ventral axial vein and mediates the absorption of nutrients from the yolk [20]. Similar to angiogenesis in the CVP region, we found that endothelial sprouting was significantly delayed in the SIVP in *slc39a5* morphants (electronic supplementary material, figure S2*a–c*), suggesting that the delayed venous angiogenesis in *slc39a5* morphants is a general phenomenon.

To confirm that the effects of the *slc39a5* morpholino were due specifically to knocking down *slc39a5*, we performed a rescue experiment in which we co-injected single-cell embryos with both the *slc39a5* morpholino and an ATG-MO-insensitive *slc39a5* mRNA containing a 5-nucleotide mismatch at the 5′ end; for these experiments, we used the transgenic *Tg*(*fli1a*:eGFP) zebrafish line in which endothelial cells express eGFP [4]. We found that expressing the ATG-MO-insensitive *slc39a5* mRNA restored the vascular structure (figure 2*j*), preventing both the reduction in endothelial sprout formation (figure 2*k*) and reduced capillary loop formation (figure 2*l*).

### 2.3. Reduced caudal vein plexus angiogenesis in *slc39a5* morphants is due to impaired proliferation and migration of endothelial cells

**Figure 3.**
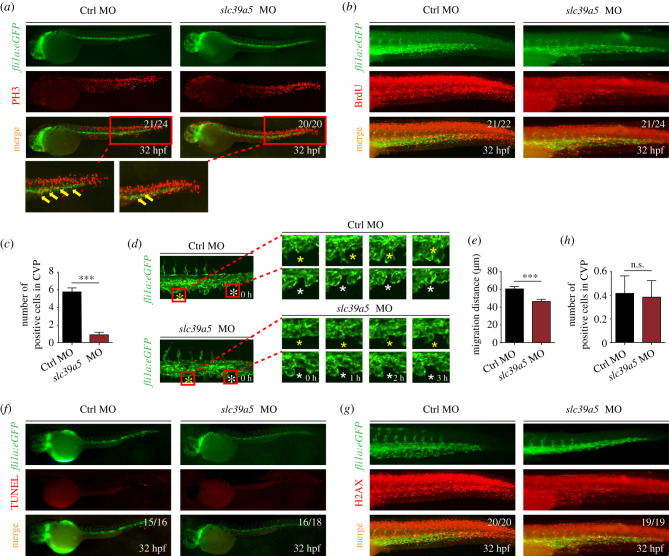
*slc39a5* morphants have impaired endothelial cell proliferation and migration in the CVP region. (*a*–*b*) PH3 (*a*) and BrdU (*b*) immunostaining of control and *slc39a5* morphant *Tg*(*fli1a*:eGFP) embryos, with magnified views of the CVP showing co-localization of GFP fluorescence and PH3 immunoreactivity (arrows). (*c*) Summary of BrdU-positive cells in the CVP of control and *slc39a5* morphants. ***p < 0.001. (*d*) Representative sequential images of the CVP in control and *slc39a5* morphant *Tg*(*fli1a*:eGFP) embryos. For each embryo, two separate endothelial cells are indicated with a white asterisk and a yellow asterisk. Note that 0 h corresponds to 32 hpf. (*e*) Summary of the migration distance of endothelial cells from 32 to 35 hpf in the CVP of control and *slc39a5* morphant *Tg*(*fli1a*:eGFP) embryos. (*f*) Representative images of TUNEL staining in the CVP of control and *slc39a5* morphant *Tg*(*fli1a*:eGFP) embryos. (*g*–*h*) Representative images (*g*) and summary (*h*) of H2AX immunostaining in the CVP of control and *slc39a5* morphant *Tg*(*fli1a*:eGFP) embryos.

During angiogenesis, sprout elongation is driven primarily by the proliferation and migration of endothelial cells [21]. Given the reduction in endothelial sprouting in *slc39a5* morphants, we measured endothelial cell proliferation in the CVP region using phosphohistone H3 (PH3) and BrdU immunostaining. We found that both PH3 and BrdU staining were significantly reduced in the caudal veins in *slc39a5* morphants (figure 3*a–c*), consistent with reduced cell proliferation in the CVP.

Next, we monitored the migration of endothelial cells in the CVP region from 32 hpf through 35 hpf using high-resolution spinning disk confocal microscopy. In control embryos, two venous endothelial cells sprouted at 33 hpf and gradually elongated to form a loop structure over the following 3 h (figure 3*d*). By contrast, endothelial cells in the same region were relatively loss mobile in the *slc39a5* morphants (figure 3*d*), with significantly reduced migration over the same 3 h window (figure 3*e*). Taken together, these data support the notion that endothelial cell proliferation and migration are drastically reduced in the absence of Slc39a5.

We also measured cell death using TUNEL staining and analysed DNA damage by measuring the accumulation of H2AX (H2A histone family member X) in embryos during angiogenesis. We found that neither cell death nor DNA damage was significantly different between the control embryos and the *slc39a5* morphants (figure 3*f–h*).

### 2.4. Knocking out *slc39a5* in zebrafish produces a similar phenotype, including impaired angiogenesis

**Figure 4.**
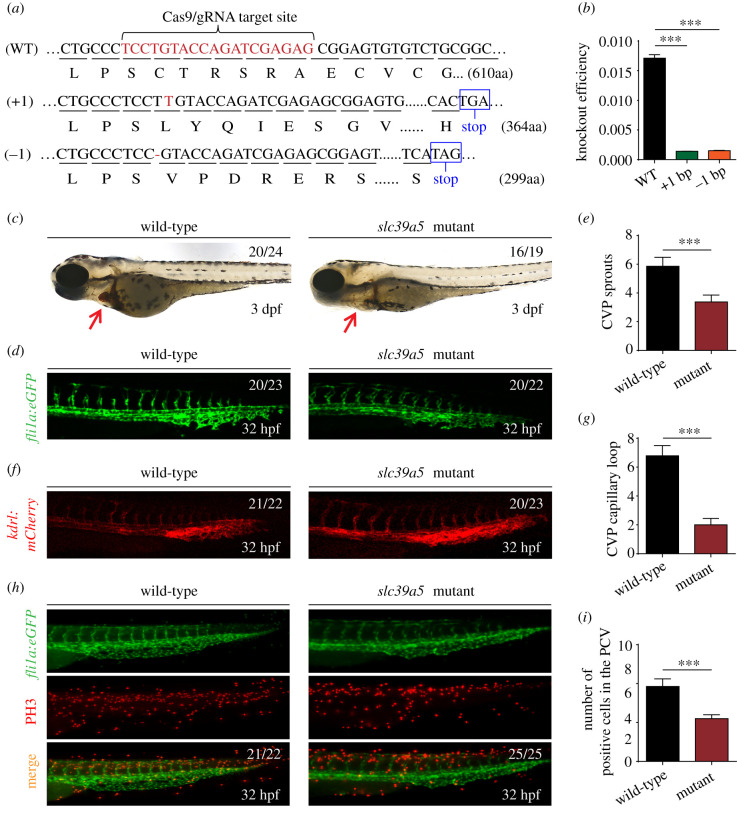
Targeting strategy and characterization of *slc39a5* knockout zebrafish. (*a*) DNA and corresponding amino acid sequences of the wild-type (WT) *slc39a5* allele and the *slc39a5* allele after inserting one nucleotide (+1) or deleting one nucleotide (−1) using CRISPR/Cas9-based editing. Both the insertion and the deletion introduce a premature stop codon. (*b*) Summary of *slc39a5* mRNA measured using qPRC in the WT, +1, and −1 *slc39a5* mutant lines (*n* = 3 sets of 50 pooled embryos/group). (*c*) Representative images of a wild-type and mutant embryo at 3 dpf. Note the significantly smaller heart with reduced cardiac blood flow in the mutant embryo (arrow). (*d*–*e*) Representative images of the CVP in wild-type (left) and mutant (right) *Tg*(*fli1a*:eGFP) (*d*) and summary of CVP sprouting in these embryos (*e*). (*f*–*g*) Representative images of the CVP in wild-type (left) and mutant *Tg*(*kdrl*:*mCherry*) (*f*) and summary of capillary loops in the CVP (*g*) in these embryos. (*h*) Representative images of PH3 immunostaining in the CVP of wild-type and mutant *Tg*(*fli1a*:eGFP) embryos. (*i*) Summary of PH3-positive cells in the CVP of wild-type and mutant *Tg*(*fli1a*:eGFP) embryos at 32 hpf. ***p < 0.001.

To confirm the role of Slc39a5 in venous angiogenesis using an independent approach, we generated *slc39a5* knockout mutants using CRISPR/Cas9 and obtained two separate lines with specific genetic mutations in the target gene; both mutations introduce a frame-shift, resulting in a premature stop codon (figure 4*a*). Quantitative real-time PCR confirmed that *slc39a5* expression is virtually abolished in both mutant lines (figure 4*b*). Consistent with our knock-down strategy, both mutant lines develop a phenotype similar to *slc39a5* morphants, with reduced blood flow in the heart (figure 4*c*), impaired angiogenesis in the CVP, delayed endothelial sprouting and fewer capillary vascular loops (figure 4*d–g*) compared with control embryos. Also consistent with our previous results, endothelial cell proliferation was significantly lower in mutant embryos compared with controls (figure 4*h*,*i*), while cell death was unaffected (electronic supplementary material, figure S3). Thus, both knocking down and knocking out *slc39a5* revealed that it plays a central role in venous angiogenesis.

### 2.5. *Slc39a5*-mediated zinc homeostasis regulates angiogenesis in the caudal vein plexus

**Figure 5.**
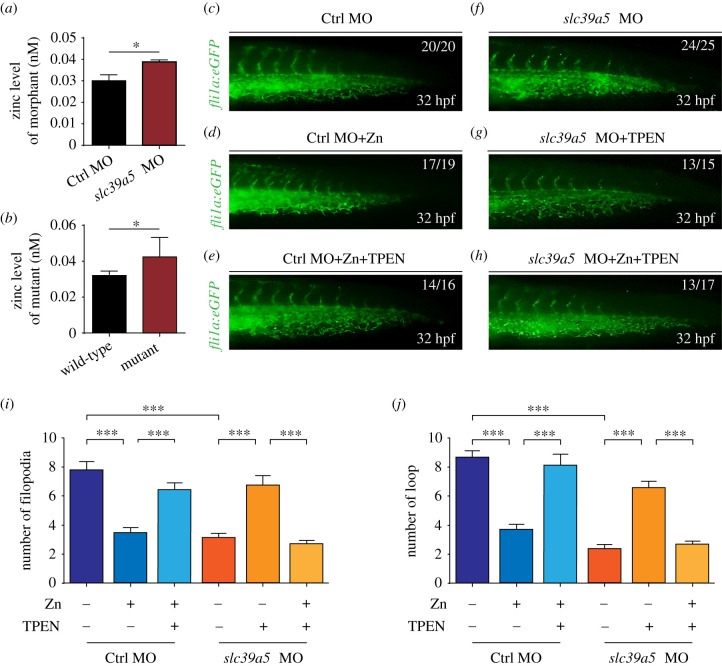
Slc39a5 regulates CVP formation by maintaining zinc homeostasis. (*a*–*b*) Summary of Zn concentration measured in control and *slc39a5* morphant embryos (*a*) and in wild-type and *slc39a5* knockout mutant embryos (*b*) at 3 dpf using the fluorescent zinc probe FluoZin-3 AM (*n* = 3 sets of 50 pooled embryos per group). (*c*–*h*) Representative images of control and *slc39a5* morphant embryos treated with 1 mM zinc solution and/or the zinc chelator TPEN. (*i*–*j*) Summary of capillary filopodia (*i*) and loops (*j*) in the CVP of control and *slc39a5* morphant embryos at 32 hpf; where indicated, the embryos were treated with zinc solution and/or TPEN.

Given that Slc39a5 is a putative zinc transporter that regulates systemic zinc concentrations [13,14], we used a fluorescent zinc probe to measure the effect of deleting Slc39a5 on systemic zinc levels in zebrafish embryos. We found that compared with control embryos, both the *slc39a5* morphants and the *slc39a5* knockout embryos had higher levels of zinc (figure 5*a*,*b*), consistent with zinc accumulation in absence of Slc39a5. Next, we examined the role of zinc homeostasis in venous angiogenesis by treating control embryos and *slc39a5* morphants with a high zinc solution (to increase systemic zinc levels) and/or the cell-permeable zinc chelator TPEN. We found that zinc-treated control embryos had reduced filopodia sprouting and fewer capillary loops (figure 5*c–d*,*i*,*j*), mimicking the phenotype observed in *slc39a5* morphants in which zinc was accumulated; moreover, these effects were absent in control embryos co-treated with both zinc and TPEN (figure 5*c*,*e*,*i*,*j*). Finally, treating *slc39a5* morphants with TPEN restored angiogenesis, with increased filopodia sprouting and capillary loop formation (figure 5*f*,*g*,*i*,*j*), while co-treatment with TPEN and zinc neutralized the ability of TPEN to rescue angiogenesis in *slc39a5* morphants (figure 5*f*,*h*,*i*,*j*). Taken together, these results indicate that loss of Slc39a5 in zebrafish embryos disrupts zinc homeostasis and impairs venous angiogenesis.

## 3. Discussion

Angiogenesis is orchestrated by a close interaction between endothelial cells and their niche [22]. Although a variety of signals and processes have been investigated with respect to their role in angiogenesis, how zinc and zinc homeostasis play a role in angiogenesis is poorly understood. Here, we report that the zinc transporter Slc39a5 plays an essential role in venous angiogenesis in zebrafish, and we report that zinc homeostasis is required for endothelial budding and the proliferation and migration of endothelial cells.

Our *in vivo* results show that *slc39a5* is widely expressed in zebrafish embryos during early development, subsequently becoming concentrated in the head and gut region. Previous studies showed that SLC39A5 localizes to the basolateral surface of enterocytes in mice fed a zinc adequate diet, suggesting that this protein may remove zinc from the blood via the intestine [13,14]. These reports support our finding that knocking down *slc39a5* leads to systemic zinc accumulation in zebrafish embryos, indicating that Slc39a5 plays an essential role in maintaining systemic zinc homeostasis and functions to remove excess zinc from the body.

Our finding that *slc39a5* morphant embryos develop a clear phenotype that includes cardiac ischaemia and the accumulation of blood in the CVP suggests the possibility of toxicity and/or an off-target effect of the morpholino [23,24]. We believe these effects are unlikely, however, as co-injecting single-cell embryos with a morpholino-resistant *slc39a5* transcript together with the *slc39a5* morpholino restored normal development, thereby preventing the phenotype induced by the *slc39a5* morpholino. In addition, we generated two separate *slc39a5* knockout lines using CRISPR/Cas9 and found that the resulting phenotype is similar to the *slc39a5* morphants, confirming that both cardiac ischaemia and blood accumulation in the CVP are due to the loss of Slc39a5.

In the condition of *slc39a5* deficiency, although the endothelial sprouting and migration in CVP and SIVP were delayed, the intersegmental vessel (ISV) showed a relative normal development (figure 2*j*; electronic supplementary material, figure S4*a*). As CVP and SIVP are formed by angiogenic sprouts from the axial vein, while ISV is formed by angiogenic sprouts from the dorsal aorta [3], we speculate that Slc39a5-mediated zinc homeostasis affects venous angiogenesis in zebrafish.

Since the CVP overlaps with caudal haematopoietic tissue (CHT), an important tissue for definitive haematopoiesis [25,26], we evaluated the definitive haematopoiesis development by analysis the expression of *cmyb*, a well-known marker gene for definitive haematopoiesis in zebrfish [27,28]. By using a transgenic fish line Tg(*cmyb*:eGFP), we found that the formation and distribution pattern of haematopoietic stem cells were not significantly changed at 2–3 dpf, while the expression of *cmyb* showed a slight increase at 4–5 dpf in *slc39a5* morphants (electronic supplementary material, figure S4*b*). *In situ* hybridization data also showed an increased *cmyb* expression at 5 dpf, which suggests more haematopoietic stem cell formation in *slc39a5* morphants (electronic supplementary material, figure S4*c*). One hypothesis to explain this phenomenon is that, since lots of red cells are accumulated in CVP, which leads to a lack of red cells in circulation, the increased haematopoietic cells in CHT could be a compensation for the insufficient blood in circulation. However, a detailed mechanism to illustrate the complex network between CVP formation and CHT haematopoiesis in the condition of *slc39a5* deficiency should be explored in the future.

Zinc is a dietary micronutrient essential for proper cellular function, and its dynamics are closely associated with cell proliferation and migration in a variety of systems and tissues, including the immune system, central nervous system and skin [7,29–31]. Moreover, *in vitro* studies showed that the proliferation and migration of human lens epithelial B3 cells are inhibited by ZnCl_2_ treatment [32], whereas the migration of human microvascular endothelial cells increases under zinc-deficient conditions [33]. To further determine whether zinc homeostasis plays a role in endothelial cell migration *in vitro*, we performed a scratch assay to detect the cell migration speed under zinc and/or TPEN treatment by using human UMbilical vein endothelial cells (HUVEC). The results showed that the cell migration speed was slowed down under zinc exposure, with significantly decreased expression of migration marker genes, including *PECAM1* and *MMP2* [34,35] (electronic supplementary material, figure S5*a–d*). Based on these data, we think that zinc homeostasis could affect the endothelium via an endothelial cell-specific manner.

In addition, a growing number of *in vivo* and *in vitro* studies suggest that zinc transporters play a role in regulating cell migration. For example, Taylor *et al*. [36] reported that knocking down *Slc39a10* delayed epiboly in the developing zebrafish embryo. Other studies using cultured cell lines found that overexpressing SLC39A9 causes increased migration of glioblastoma cells [37], and increased expression of SLC39A6 has been associated with increased intracellular levels of zinc and increased tumour invasiveness, while knocking down *SLC39A6* reduces the proliferation of oesophageal squamous cell carcinoma cells [38]. Although both cell proliferation and migration—two processes that have been studied extensively in angiogenesis—are triggered by a multitude of signals and guidance cues leading to proper endothelial cell path finding [39–41], the precise role of zinc and zinc transporters in regulating angiogenesis remains poorly understood.

**Figure 6.**
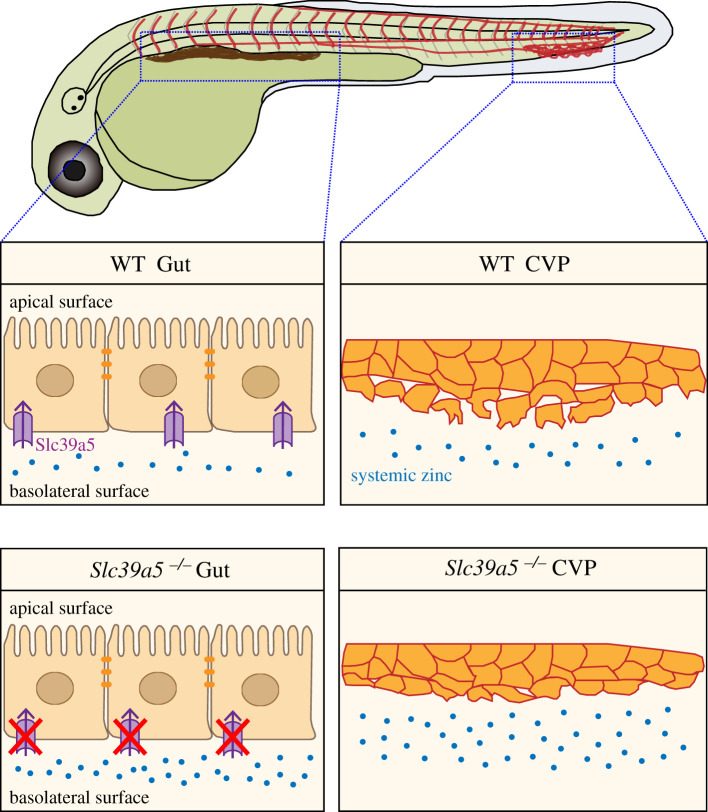
Proposed model illustrating the role of Slc39a5 in regulating systemic zinc homeostasis during angiogenesis. In wild-type embryos (top row), the zinc transporter Slc39a5 at the basolateral surface of enterocytes removes excess zinc from the circulation (top left panel; Wang *et al.* [13] and Dufner-Beattie *et al.* [14] carefully analysed the Slc39a5 location in polar cells), maintaining systemic zinc homeostasis and supporting venous angiogenesis in the CVP (top right panel); in the absence of Slc39a5 (bottom row), zinc accumulates in the embryos (bottom left panel), impairing venous angiogenesis in the CVP (bottom right panel).

In summary, our results support a model in which Slc39a5-mediated zinc homeostasis is essential for the normal development of venous angiogenesis, as loss of Slc39a5 results in systemic zinc accumulation, delayed CVP sprouting and impaired endothelial cell migration (figure 6). These findings provide insights into the way in which zinc regulates the microenvironment during angiogenesis, suggesting potential new molecular targets for the study and treatment of angiogenesis-related physiological and pathological conditions.

## 4. Material and methods

### 4.1. Zebrafish

Zebrafish were raised and maintained in accordance with guidelines established by the Laboratory Animal Center, Zhejiang University. Transgenic zebrafish lines were purchased from the Chinese Zebrafish Resource Center in Wuhan, China.

### 4.2. Whole-mount *in situ* hybridization

The 1-kb antisense probe was cloned into pEASY-T3 (TransGen Biotech), and the sense and antisense probes were synthesized using T7 or SP6 RNA polymerase (TR101-02, Vazyme; AM1340, mMESSAGE mMACHINE). *In situ* hybridization was performed essentially as previously described [42], and images were captured using a Nikon SMZ18 stereo microscope.

### 4.3. Microinjection of morpholinos and mRNA

Morpholinos (0.5 mM, GeneTools) and/or mRNA (200–300 pg/embryo) were injected into embryos in the single-cell stage. The sequence for the morpholinos were: *slc39a5* 5′-AGACTGTCTGTCCACCCATGATTTT-3′; control 5′-CCTCTTACCTCAGTTACAATTTATA-3′. mRNA was synthesized using the mMESSAGE mMACHINE SP6 transcription kit (AM1344, Ambion) and extracted and purified using the Quick-RNA MicroPrep kit (R1050, Zymo Research).

### 4.4. Generation of knockout zebrafish lines using CRISPR/Cas9

The Cas9/gRNA target site was designed and selected from the CHOPCHOP website (https://chopchop.cbu.uib.no/). Primers containing the T7 or SP6 promoter sequences were synthesized and used for amplification from a gRNA template (pMD19-gRNA scaffold plasmid) [43]. gRNAs were transcribed *in vitro* using T7 or SP6 RNA polymerase. Cas9 protein (M0646T, New England Biolabs) and gRNA were mixed and then injected into single-cell zebrafish embryos, and T7 endonuclease 1 enzyme was used to evaluate the efficiency of target gene disruption. F0 embryos with the highest editing efficiency were raised to sexual maturity, and heterozygous F1 fish were obtained and identified from the offspring of F0 fishes using DNA sequencing. F1 fish carrying the same mutation were crossed in order to generate F2 homozygous mutant fish.

### 4.5. o-Dianisidine staining

An o-Dianisidine stock solution containing 100 mg o-Dianisidine dissolved in 70 ml ethanol was stored at 4°C in the dark. For staining, live embryos were exposed to the o-Dianisidine working solution consisting of 2 ml o-Dianisidine stock solution, 0.1 M sodium acetate (pH 4.5) and 0.65% H_2_O_2_ in the dark for 3–5 min at room temperature; images were captured using a Nikon SMZ18 stereo microscope (Nikon, Japan).

### 4.6. Zinc staining

The fluorescent zinc indicator FluoZin-3 AM (F24195, Invitrogen) was used to measure intracellular zinc concentration as reported previously [44]. Embryos were anaesthetized, ground thoroughly in phosphate-buffered saline (PBS), and then centrifuged to remove the PBS. The cells were then suspended in 200 µl detection buffer consisting of 5 mM glucose, 1 mM MgCl_2_, 1 mM NaH_2_PO_4_, 1.3 mM CaCl_2_, 25 mM HEPES, 120 mM NaCl, 5.4 mM KCl and 1 µM FluoZin-3 AM (pH 7.5), and incubated at 37°C for 30 min. After washing several times with detection buffer, the cells were resuspended in 1 ml detection buffer and divided equally into three sample tubes. Tube 1 was used to measure the average fluorescence value (F); 5 µl zinc sulfate solution (10 mM, Sigma) and 5 µl NaPr cell membrane penetrating solution (Sigma) were added to tube 2 and used to measure maximum fluorescence (*F*_max_); finally, 7.5 µl TPEN solution (2 mM, Sigma) was added to tube 3 and used to measure minimum fluorescence (*F*_min_). Each sample was then adjusted to 500 µl with detection buffer, filtered through a 350 μm mesh filter into the flow tube, and analysed using flow cytometry. The following formula was then used to calculate the zinc concentration [Zn]: *K*_D_ × [(*F* − *F*_min_)/(*F*_max_ − *F*)], where *K*_D_ is 15 (nM).

### 4.7. Immunofluorescence and TUNEL assay

The TUNEL assay was performed using the fluorescein-based Roche In Situ Cell Death Detection Kit (12156792910, Roche) in accordance with the manufacturer's instructions. For PH3 immunostaining, anti-pH3-Ser-10 (sc-8656-R, Santa Cruz) and Cy3-conjugated goat anti-rabbit (A0516, Beyotime) were used as the primary and secondary antibodies, respectively. For H2AX immunostaining, anti-phospho-Histone H2A.X (Ser139) (05-636-I, EMD Millipore) and Cy3-conjugated goat anti-rabbit (A0516, Beyotime) were used as the primary and secondary antibodies, respectively. BrdU immunostaining was performed using the BrdU Labeling and Detection Kit (10280879001, Roche) in accordance with the manufacturer's instructions.

### 4.8. Reverse transcription and quantitative PCR

More than 30 embryos were anaesthetized and transferred to TRIzol Reagent (Ambion) for total RNA extraction. the PrimeScript RT Kit (RR047A, Takara Bio) was then used to synthesize cDNA using a standard protocol, and 2× SYBR Green qPCR Master Mix (B21202, Bimake) was used for real-time PCR. Target gene expression was normalized to *ef1α* mRNA.

### 4.9. Spinning disk confocal live-cell imaging

Live embryos were embedded carefully in soft agarose (5265, TaKaRa), and images were captured in the time-lapse mode using a SpinSR10 spinning disk confocal super-resolution microscope (Olympus).

### 4.10. Quantification and statistical analysis

All experiments were performed using at least three biological replicates. Statistical analyses were performed with Prism (GraphPad) using a two-tailed Student's *t*-test, and differences with a *p*-value < 0.05 were considered statistically significant.

### 4.11. Scratch assay

HUVEC were seeded in 12-well plates. After 24 h, a straight scratch wound was made by a sterilized 10 µl pipette tip and then washed by PBS. Then the cells were cultured in fresh medium for 12 h. Cells were washed by PBS and stained by Crystal Violet Staining Solution (0.2% solution dissolved in 10% ethanol) at 0, 6 and 12 h. Images of the scratch area were captured under an inverted microscope (Olympus, Japan).

## Supplementary Material

Figures S1 - S5

## Data accessibility

This article has no additional data.
