# Supplementary material for: Slc39a5-mediated zinc homeostasis plays an essential role in venous angiogenesis in zebrafish
Source: Open Biol. 2020 Oct 21;10(10):200281. doi: 10.1098/rsob.200281 (PMC7653363; doi:10.1098/rsob.200281)
Supplement: Figures S1 - S5 [file rsob200281supp1.docx]

**ELECTRONIC SUPPLEMENTARY MATERIAL**

**Slc39a5-mediated zinc homeostasis plays an essential role in angiogenesis in zebrafish**

Zhidan Xia^1^, Xinying Bi ^1^, Jia Lian^1^, Wei Dai^1^, Xuyan He^1^, Lu Zhao^1^, Junxia Min^1^, Fudi Wang^1*^

^1^The First Affiliated Hospital, School of Public Health, Institute of Translational Medicine, Zhejiang University School of Medicine, Hangzhou, China

*Open Biology*, Manuscript RSOB-20-0281


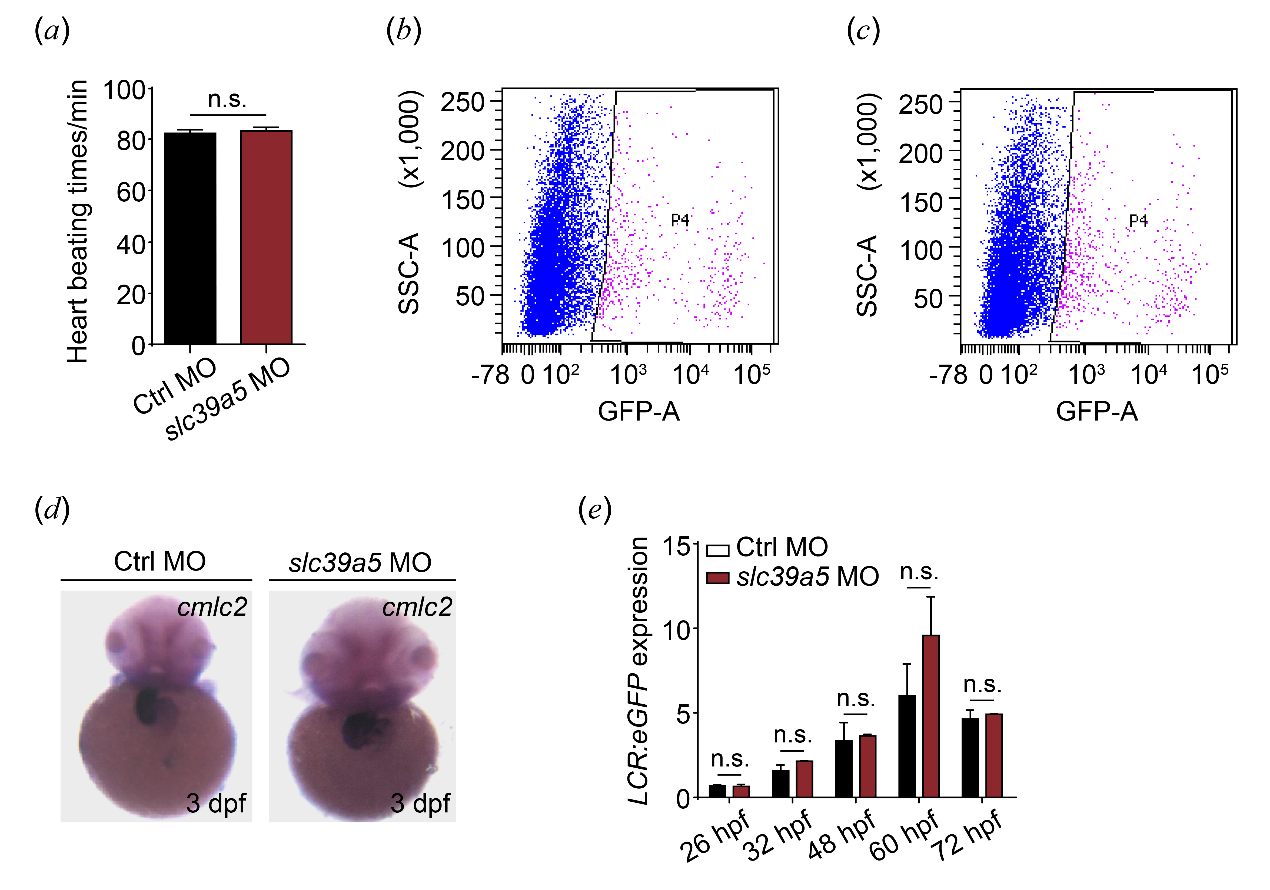


**Figure S1. Cardiac morphology and summary of red blood cells in control and** ***slc39a5* morphant embryos during development.**

(*a*) Summary of the heart rate measured in control and *slc39a5* morphant embryos.

(*b*) Example images of *cmlc2* mRNA measured in control and *slc39a5* morphant embryos using whole-mount *in situ* hybridization.

(*c-d*) Cell sorting experiments of RBCs isolated from control (*c*) and *slc39a5* morphant (*d*) *Tg(globinLCR:eGFP)* embryos, with GFP fluorescence on the *x*-axis and side scatter on the *y*-axis. The region marked “P4” indicates the gating strategy used for the summary data presented in panel E.

(*e*) Summary of the percentage of GFP-positive RBCs measured in control and *slc39a5* morphant *Tg(globinLCR:eGFP)* embryos at the indicated stages of development.

n.s., not significant (*P*>0.05).

**
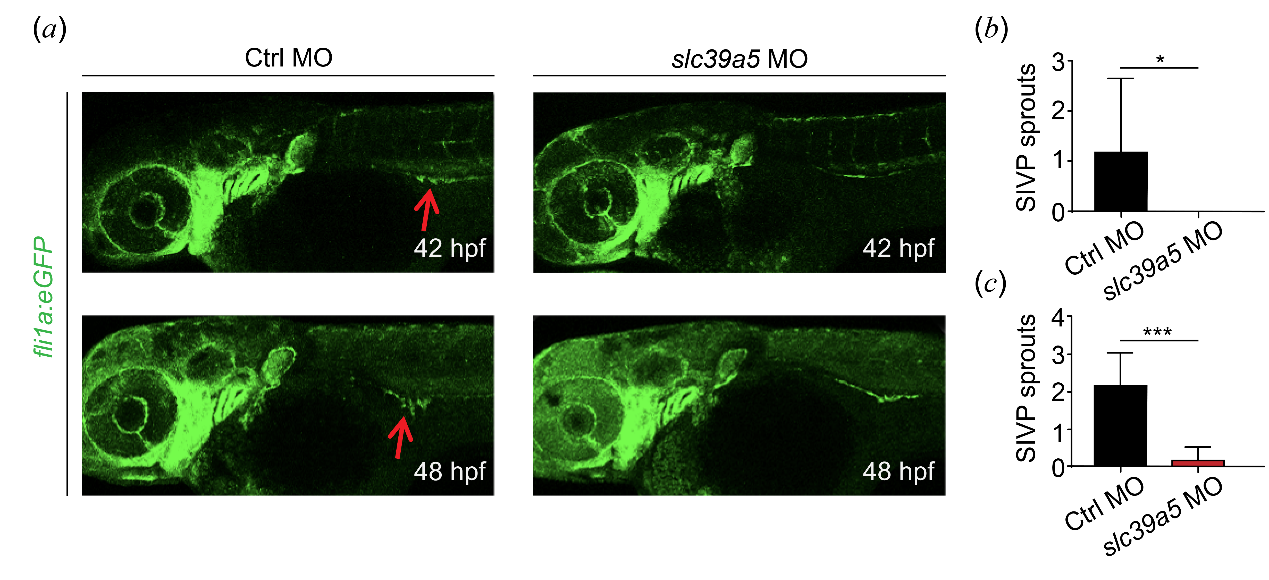
**

**Figure S2. Angiogenesis is impaired in the sub-intestinal vein plexus of *slc39a5* morphant embryos.**

(*a*) Representative images of the SIVP in control and *slc39a5* morphant *Tg(fli1a*:eGFP*)* embryos at 42 hpf (top row) and 48 hpf (bottom row). The red arrows indicate sprouts in the SIVP region.

(*b*-*c*) Summary of endothelial sprouts measured in the SIVP region of control and *slc39a5* morphant *Tg(fli1a*:eGFP*)* embryos at 42 hpf (*b*) and 48 hpf (*c*).

**P*<0.05 and ****P*<0.001.


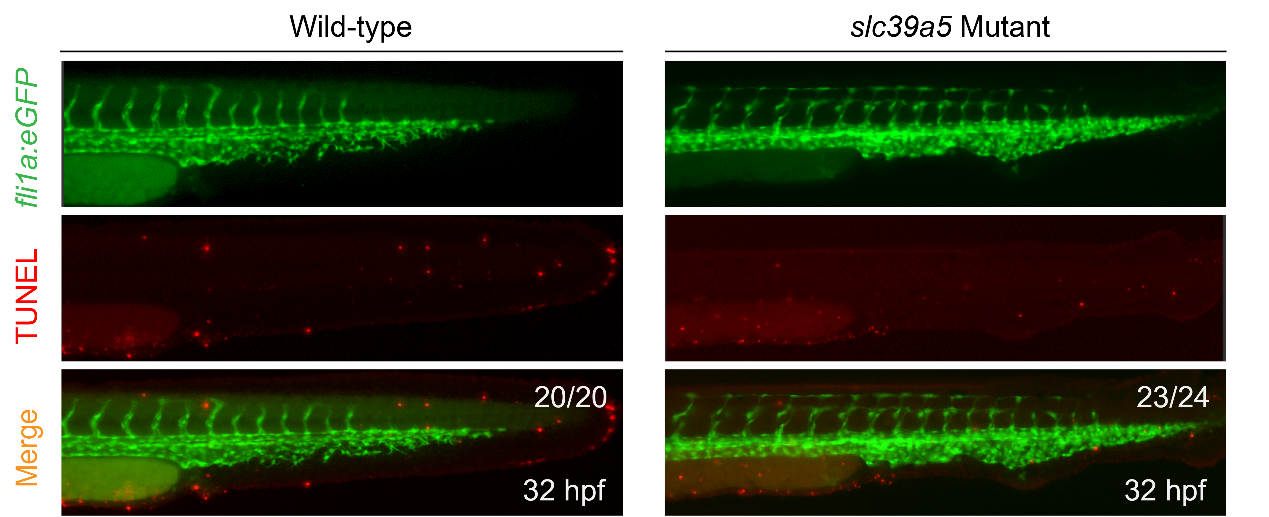


**Figure S3. TUNEL assay showing no difference in apoptosis in the CVP between wild-type and *slc39a5* knockout *Tg(fli1a*:eGFP*)* embryos.**

**
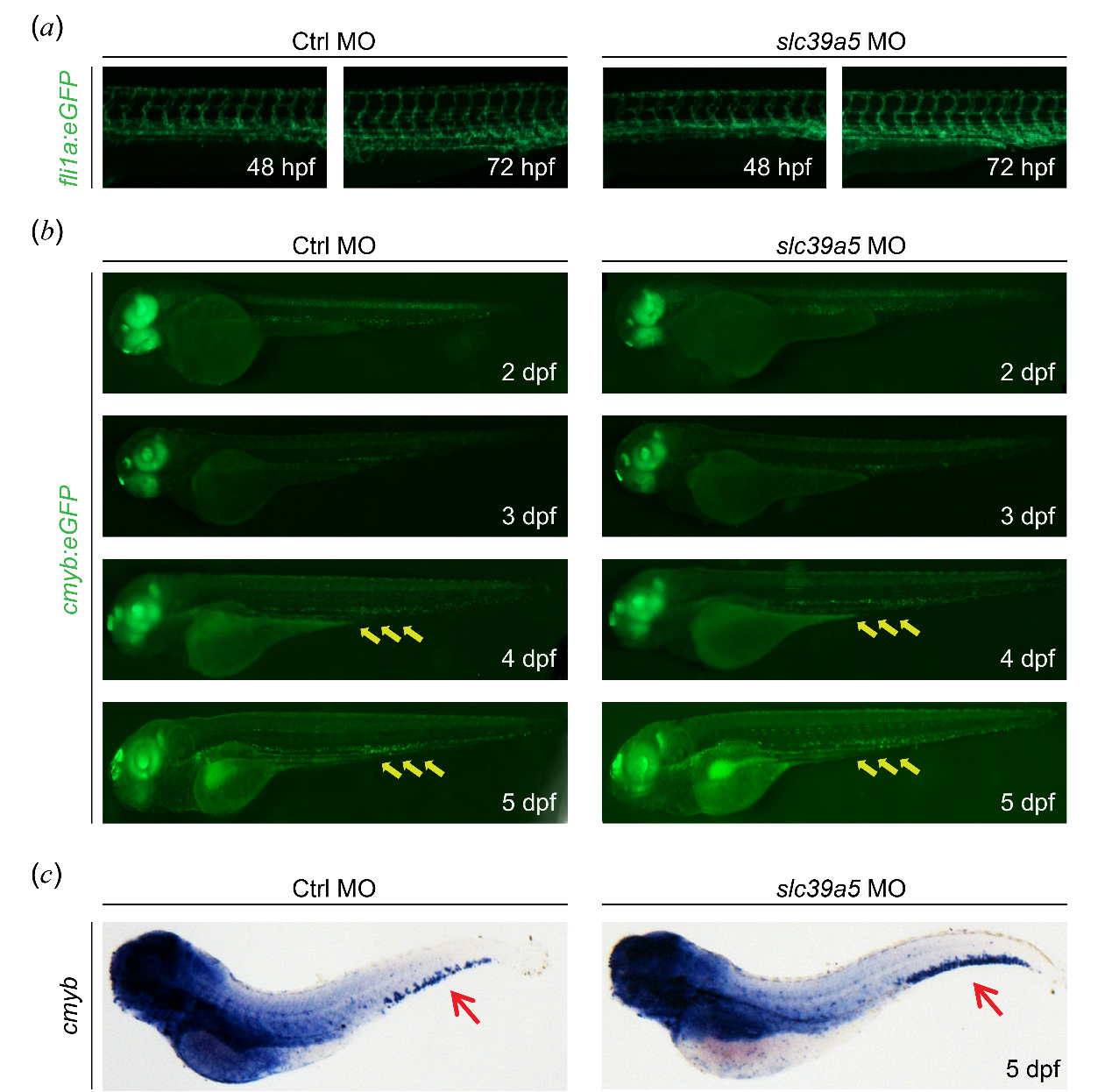
**

**Figure S4. Increased hematopoietic cells detected in *slc39a5* morphants.**

(*a*) Representative images of the ISV in control and *slc39a5* morphant *Tg(fli1a*:eGFP*)* embryos at 48 hpf and 72 hpf.

(*b*) Representative images of the control and *slc39a5* morphant *Tg*(*cmyb*:eGFP) embryos. Note the slightly increased number of the hematopoietic stem cells (yellow arrows) in *slc39a5* morphants.

(*c*) Whole-mount *in situ* hybridization of *cmyb* in control and *slc39a5* morphants, the red arrows indicate hematopoietic stem cells.


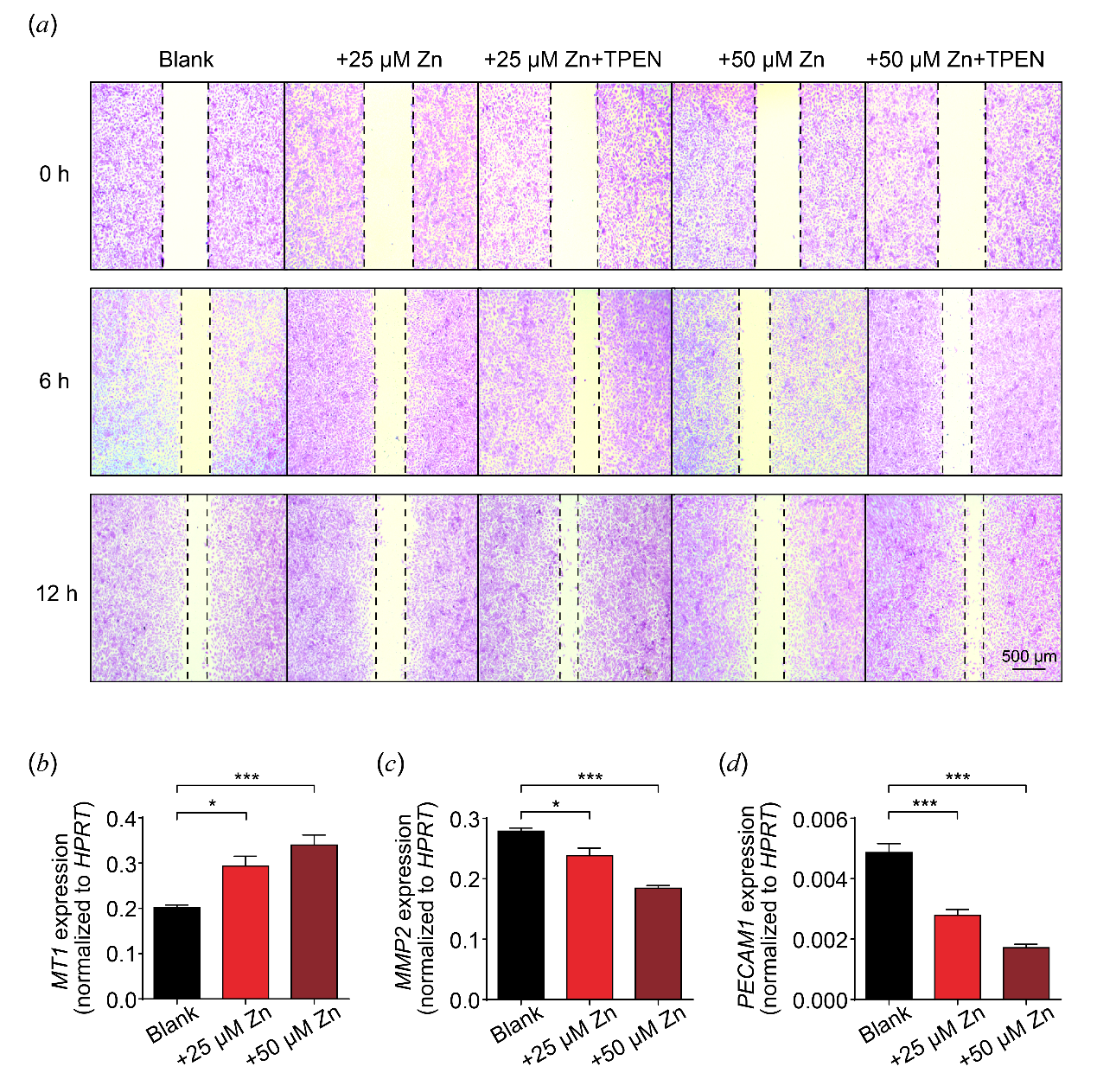


**Figure S5. Cell migration detected using HUVEC.**

(*a*) Representative images of HUVEC scratch area at 0 h, 6 h and 12 h; where indicated, the cells were treated with zinc solution and/or TPEN. Note the cell migration speed was slow down under zinc exposure.

(*b-d*) Summary of *MT1* mRNA (*b*), *MMP2* mRNA (*c*), and *PECAM1* mRNA (*d*) in HUVEC treated with 25 μM zinc or 50 μM zinc.

**P*<0.05 and ****P*<0.001.
